# Proton pump inhibitor effect on macrophage and neutrophil function: a systematic review

**DOI:** 10.3389/fimmu.2024.1477993

**Published:** 2024-12-24

**Authors:** Josef F. Fowler, Taryn A. Eubank, Kevin W. Garey

**Affiliations:** College of Pharmacy, University of Houston, Houston, TX, United States

**Keywords:** omeprazole, infection, myeloid phagocyte system, innate immune response, Toll-like receptor, chemotaxis, cytokine profile

## Abstract

**Background:**

Proton pump inhibitors (PPIs) are one of the most used drugs worldwide. While generally considered safe, the usage of PPIs is associated with several adverse outcomes including acute infectious diseases. PPIs influence macrophage and neutrophil function although a systematic review has never been undertaken. The purpose of this systematic review was to determine the potential mechanisms of how PPI-induced inhibition of macrophage and neutrophil function may increase infection risk in susceptible hosts.

**Methods:**

A database search using Scopus and PubMed was performed to identify studies that investigated the effects of PPIs on neutrophils or macrophage function.

**Results:**

The final screening yielded 21 English-language research articles that focused on the impacts of PPIs on the function of macrophages and neutrophils. PPI mechanistic effects included cytotoxic effects on polymorphonuclear neutrophils, inhibition of reactive oxygen species (ROS) and reactive nitrogen species, phagocytosis and phagosomal degradation, inhibition of chemotaxis and migration, altering Toll-like receptor signaling and p38 protein phosphorylation in immune cells, and altering neutrophil and macrophage gene expression.

**Discussion:**

The impact of PPIs on MΦs and neutrophils regarding their role in the immune response to bacterial pathogens was summarized. PPI effects on macrophages and neutrophils occurred due to the therapeutic mechanism of PPIs, the protonation of sulfhydryl groups and the subsequent formation of a disulfide bond, and other pleiotropic manners. Given the common use of PPIs, these results highlight the necessity to optimize PPI use and stewardship to curtail unnecessary drug use.

## Introduction

Proton pump inhibitors (PPIs) are one of the most used drugs worldwide. Omeprazole, the most prescribed of the six available PPIs, was the eighth most prescribed drug in America in 2020, with over 56 million prescriptions (1). Worldwide, nearly a quarter of adults over 18 years of age use PPIs. Despite the FDA recommendation of a 4–8-week treatment period for PPIs (2), nearly 7% of PPI users continue therapy for more than 3 years, surpassing the recommended FDA usage guidelines and increasing the propensity for adverse long-term effects (3, 4). While generally considered safe, long-term usage of PPIs is associated with several adverse outcomes including deficiencies in calcium, magnesium, and B_12_ and increased rates of infectious diseases such as *Clostridioides difficile* infections (CDI) and community-acquired pneumonia (CAP) and neurological disorders such as dementia and Alzheimer’s disease (2, 4–7). The mechanisms underlying these risks are largely undefined; however, the increase in adverse effects, such as infection, occurring with prolonged use suggests that these effects may result from alterations to cell populations and functional capacity. The innate immune system maintains a healthy microbiome and prevents colonization by pathogenic bacteria. A deleterious effect of PPIs on macrophages (MΦs) and neutrophils would provide a causal link between their long-term use and observed adverse effects.

Macrophages (MΦs) and neutrophils are crucial cell types in the generation of effective immune responses, particularly in response to bacterial infection. These cells are involved directly through phagocytosis and the generation of oxidative radicals and enhance recruitment through the generation of inflammatory cytokines. Macrophage and neutrophil dysfunction is commonly associated with increased infections, chronic inflammation, and the inability to mount an effective adaptive immune response (8). PPIs have been shown to influence macrophage and neutrophil function although a systematic review has never been undertaken. The purpose of this systematic review was to determine the potential mechanisms of how PPI-induced inhibition of macrophage and neutrophil function may increase infection risk in susceptible hosts.

## Methods

A database search using Scopus and PubMed was performed up to 26 April 2024 using the following search terms: “*proton pump inhibitors*” AND “*macrophages*” or “*proton pump inhibitors*” AND “*neutrophils.*” Reviews, commentaries, opinions, meta-analyses, and systematic reviews were excluded. Articles kept for review were selected if PPI exposure impacted healthy macrophages and neutrophils and the articles described the contributing mechanisms. Studies where infections were established prior to PPI exposure were excluded to focus on the impacts of PPIs on the innate immune system and the mechanism behind the observed associations of increased infection risk. Two investigators (JF and TE) screened the identified articles for inclusion/exclusion with all investigators providing input on any articles in question. This systematic review was conducted and reported in accordance with the Preferred Reporting Items for Systematic Reviews and Meta-Analyses (PRISMA) guidelines.

## Results

**Figure 1:**
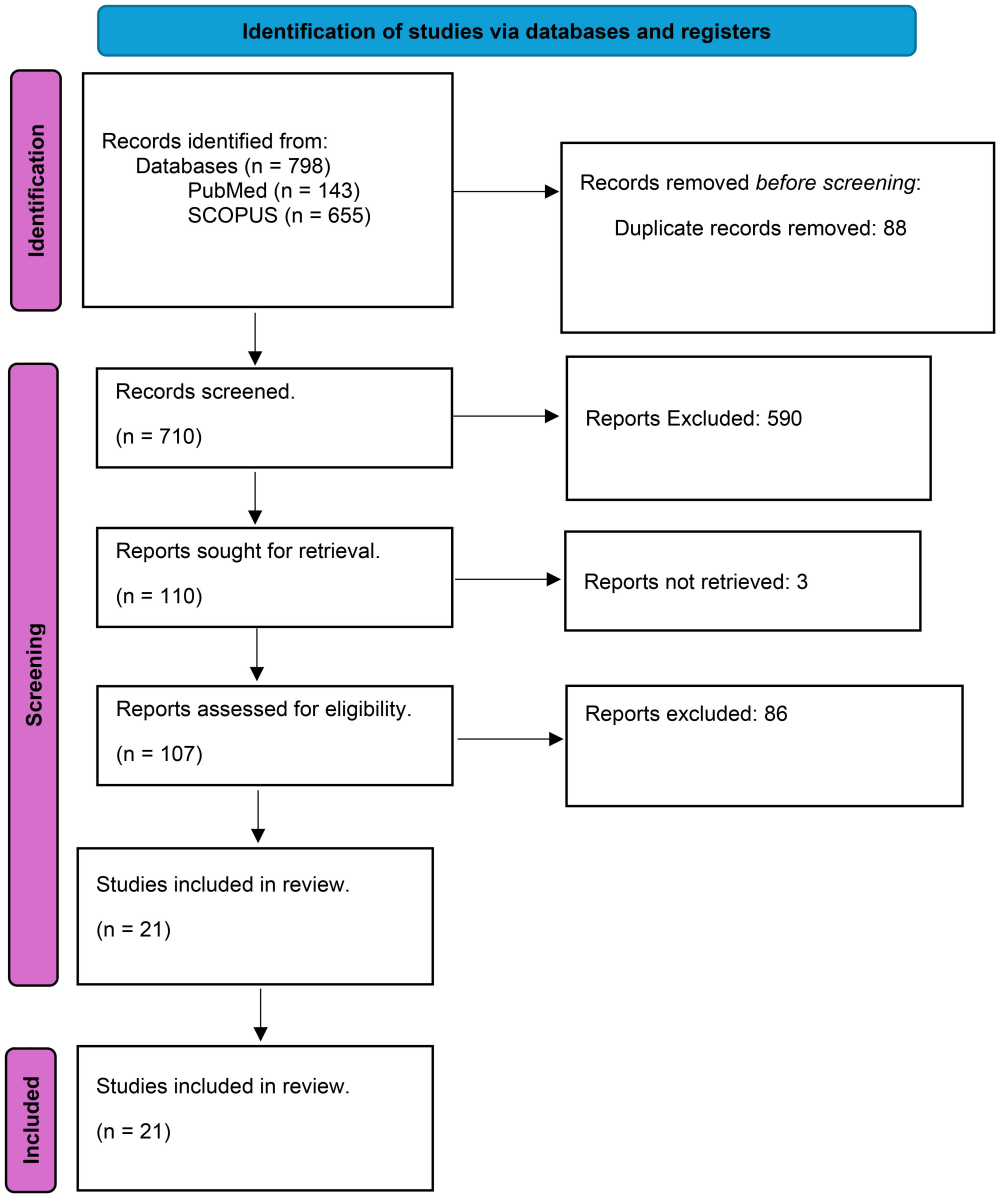
The PRISMA flowchart.

Seven hundred and ninety-eight articles were identified with 88 duplications between the two databases. After applying the inclusion and exclusion criteria, the final screening yielded 21 English-language research articles that focused on the impacts of PPIs on the function of macrophages and neutrophils (PRISMA flowchart shown in **Figure 1**; **Supplementary Table 1**). The included articles were grouped on the proposed mechanistic impact from PPI exposure. PPI mechanistic effects included cytotoxic effects on polymorphonuclear neutrophils (PMNs), inhibition of reactive oxygen species (ROS) and reactive nitrogen species (RNS), phagocytosis and phagosomal degradation, inhibition of chemotaxis and migration, altering Toll-like receptor (TLR) signaling and p38 protein phosphorylation in immune cells, and altering neutrophil and macrophage gene expression.

### PPI cytotoxic effects on polymorphonuclear neutrophils

Capodicasa et al. (9) investigated the cytotoxic effects of omeprazole (OME) and OME-HCl (hydrochloric acid) on PMNs. PMNs were isolated from 15 healthy donor blood samples incubated with OME (unacidified or acidified) at a concentration range of 1 × 10^−4^ to 2.5 × 10^−5^ for 1–14 h. After 10 h of incubation, both activated and prodrug OME induced apoptosis of neutrophils. The prodrug OME-induced apoptosis was reduced via caspase-3 inhibition, whereas activated OME-induced apoptosis was reduced via both caspase-3 and caspase-8. Pantoprazole (PAN) is also capable of inducing caspase-3-dependent cell death by enhancing mitochondrial stress because of proteasomal inhibition and unfolded protein accumulation (10).

### PPI inhibition of reactive oxygen species and reactive nitrogen species

Wandall (11) used PMNs from healthy donors exposed for 30 min to unacidified or HCl-acidified OME at concentrations from 1 × 10^−3^ to 5 × 10^−5^ to assess chemotaxis, O_2_^−^ generation, and degranulation. Preincubation of PMNs with unacidified or acidified OME reduced chemotaxis and degranulation in a dose-dependent manner compared to untreated PMNs. However, only acidified OME was shown to inhibit the generation of O_2_^−^ with a half-maximal inhibitory concentration (IC_50_) of 2.5 × 10^−6^. Suzuki et al. (12) used PMNs from 10 healthy human subjects given OME 20 mg/day for 7 days to measure ROS production. A dose-dependent reduction in oxidative radicals following stimulation with both N-formylmethionine-leucyl-phenylalanine (fMLP) and zymosan was observed. The intralysosomal pH also demonstrated a dose-dependent increase following OME exposure and fMLP or zymosan stimulation. Capodicasa et al. (13) used PMNs from 26 healthy volunteers to measure the effects of lansoprazole (LAN) on chemotaxis and O_2_^−^ generation. Following a 30-min exposure to non-acidified LAN concentrations from 50 to 1,000 μM, a dose-dependent inhibition in chemotactic movement and superoxide production was observed. Zedtwitz-Liebenstein et al. (14) used blood samples from 10 healthy subjects at baseline and then 4 h after receiving OME 40 mg. Neutrophils were analyzed for their capacity to phagocytose *Escherichia coli* and the generation of ROS and intracellular calcium concentrations were measured. Phagocytosis of *E. coli* was not impaired after OME treatment, but the destruction of phagocytosed bacteria was significantly reduced. While basal extracellular ROS production was unaffected by OME, intra- and extracellular ROS production was reduced after stimulation, and cytosolic calcium increased. Nakagawa et al. (15) used RAW264.7 murine MΦs treated with LAN 0–400 µM for 2 h before a 12-h incubation with lipopolysaccharide (LPS) 1 µg/mL to assess P-ATPase mRNA, intracellular and extracellular ROS, NADPH oxidase activity, cyclooxygenase-2 (COX-2), inducible nitrous oxide synthase (iNOS), and prostaglandin E_2_ (PGE_2_) expression. Nitrous oxide (NO) and PGE_2_ production was inhibited in a dose-dependent manner. Decreased NO production was associated with NADPH oxidase (NOX) inhibition suggesting a link between NO production and ROS, a reactive species produced by NOX. LAN inhibited ROS production following stimulation with LPS in a NADPH-oxidase-dependent manner. Since LAN inhibited the stimulation of NO induced by LPS, but not NO production by xanthine plus xanthine-oxidase (X/XO), a NADPH-oxidase-independent pathway, the authors conclude that LAN directly inhibits NADPH oxidase. Taken together, these studies demonstrate the OME inhibitory effects on the generation of ROS. ROS downregulation was observed with an increase in intracellular Ca^2+^. The mobilization of intracellular calcium plays an important role in ROS generation within the lysosome (16) as well as phagocytosis as it drives the polymerization of actin and the ingestion of extracellular components (17). These findings suggest that OME does not exert its effects through inhibition of Ca^2+^ signaling and Ca^2+^ mobilization, which conflicts with the results observed in Handa et al. (18) and Martins De Oliveira et al. (19).

### Inhibition of phagocytosis and phagosomal degradation by PPIs

Agastya et al. (20) used neutrophils isolated from three healthy subjects and exposed the neutrophils to non-acidified OME prodrug or acidified active OME, both at 0.5 mM. The treated neutrophils incubated with *Saccharomyces cerevisiae* for 10–60 min decreased total yeast phagocytosed with activated but not prodrug OME, but both prodrug OME and activated OME inhibited phagolysosome acidification. Haas et al. (21) used peripheral blood mononuclear cells (PBMCs) and granulocytes from 30 healthy volunteers (12 receiving PPIs and 18 not receiving PPIs) to assess the impact of PAN on PMN function. PMNs and whole blood samples were incubated with PAN 100 µM for 30 min. PAN inhibited interleukin (IL)-2, IL-6, tumor necrosis factor-α (TNF-α), and interferon-γ (IFN-γ) and decreased phagocytosis, oxidative burst, and fMLP-induced migration. PMNs treated with prodrug PAN did not inhibit phagolysosomal acidification as demonstrated by increased levels of red fluorescence of *DsRed E. coli* or pHrodo Red *E. coli* particles when compared to bafilomycin A-treated cells. Bosnjak et al. (22) used RAW264.7 MΦs treated with LAN (range 1–10,000 nM/L) for 48 h to show concentration- and pH-dependent inhibition of the lysosomal cysteine proteases legumain and cathepsin B. LAN had no direct effect on phagolysosome acidification. Taken together, these studies demonstrate PPI inhibition of phagocytosis and phagosomal degradation. The acidification of lysosomes and phagolysosomal fusion are both dependent on V-ATPase function (23). These V-ATPases have been shown to be inhibited by disulfide bond formation (24), the same bond type that PPIs form to inhibit H^+^/K^+^ ATPases on parietal cells (25). Although Bosnjak et al. (22) did not find that treatment with LAN inhibited the acidification of lysosomal compartments, they did report that LAN directly inhibits the cysteine proteases cathepsin B and legumain through a direct interaction. This interaction was abolished through the addition of dithiothreitol and glutathione, reducers of disulfide bonds, suggesting that the inhibitory mechanism is the formation of a disulfide bond between the sulfhydryl group of LAN and the catalytic cysteine of the proteases.

### Inhibition of chemotaxis and migration by PPIs

Ritter et al. (26) used PMNs from healthy subjects to assess 30-min exposure to OME 10 μM on cell volume, intracellular pH, chemotaxis, and bacterial clearance. OME inhibited random and fMLP-induced migration and fMLP-induced cell swelling but did not inhibit bacterial clearance. Ohara and Arakawa (27) used PBMCs from 10 healthy subjects following a 2-day treatment with LAN 30 mg/day collected 7, 14, and 21 days after the start of treatment to show LAN-dependent decreased monocyte count and the number of cells expressing intracellular adhesion molecule (ICAM)-1. These alterations were not observed in cells treated with the H-2 inhibitor ranitidine, indicating a specificity for the PPI, LAN, in this mechanism. Yoshida et al. (28) used neutrophils from healthy adult men and treated the cells for 20 min with OME or LAN 10^−6^ to 10^−4^ mol/L followed by exposure to *Helicobacter pylori* water extract (HPE) or IL-1β. LAN and OME inhibited adhesion between neutrophils and epithelium following stimulation with HPE or IL-1β and decreased the expression of CD11b/CD18 and ICAM-1 in neutrophils. Haas et al. (21) used neutrophils isolated from healthy adult men. Cells were pretreated with OME or LAN 10^−4^ to 10^−7^ mol/L for 4 h prior to IL-8 or fMLP stimulation. LAN and OME were shown to inhibit the trans-endothelial migration of PMNs following stimulation with IL-8. LAN, but not OME, was shown to inhibit the increase in intracellular calcium that followed stimulation with fMLP. Martins De Oliveira et al. (19) used neutrophils collected from healthy donors and then exposed the cells to OME or PAN 10^−6^ to 10^−4^ mol/L to measure chemotaxis, cytotoxicity, and calcium mobilization. OME and PAN had no toxic effects on neutrophils. When IL-8 was added to PPI-treated cells, migration was inhibited which was reversed using nigericin, a K^+^ ionophore. PPI pretreatment reduced cytoplasmic calcium and p38 mitogen-activated protein kinase (MAPK) phosphorylation after stimulation of fMLP. Taken together, treatment with PPIs inhibits chemotactic migration and leukocyte recruitment likely via alteration of signaling and gene expression.

### PPIs alter TLR signaling and p38 protein phosphorylation in immune cells

Koshio et al. (29) isolated PMNs from healthy adult donors and exposed the cells to LAN 1–5 µg/mL for 1 h. Cells were stimulated with IL-8, fMLP, or phorbol myristate acetate (PMA) to assess p38 MAPK and extracellular signal-regulated kinase 1/2 (ERK1/2) phosphorylation. LAN increased p38 MAPK phosphorylation (phospho-38) in a dose-dependent manner, but the effect was reduced in concentrations above 25 µg/mL. LAN treatment did not directly inhibit ERK1/2 and p38 MAPK phosphorylation, or ERK1/2 induced by IL-8, but inhibited the phosphorylation of ERK2 induced by PMA and the phosphorylation of p38 MAPK induced by fMLP. Balza et al. (30) used monocytes collected from four healthy donors and incubated the cells with OME 300 µM with LPS, zymosan, or both to assess the impact of OME on the secretion of IL-1β and TNF-α induced by pathogen-associated molecular patterns (PAMPs; LPS or zymosan). OME inhibited the secretion of IL-1β and TNF-α by 80% following LPS stimulation (IC_50_: 100–300 µM). Monocytes were also collected from patients with cryopyrin-associated periodic syndrome (CAPS), an inflammatory autoimmune disease, to assess the impact of OME on IL-1β secretion. Treatment with OME also led to an 80% reduction in IL-1β secretion. The OME effect was not specific for TLR4 (receptor for LPS), as IL-8 was also OME-inhibited after stimulation by R848 (resiquimod) recognized by TLR7/8 and zymosan recognized by TLR2. OME did not directly inhibit IL-1β or TNF-α transcription or translation; rather, it inhibited K^+^ efflux, thus inhibiting the nucleotide-oligomerization domain (NOD)-like receptor (NLR) family pyrin domain-containing 3 (NLRP3) inflammasome assembly and caspase-1 activation preventing the generation of mature IL-1β.

The effects of esomeprazole (ESO) on inflammation were measured using a murine model of acute toxic shock sepsis. In the study of Balza et al. (30), mice were administered ESO 30 min before or after being given a lethal dose of LPS (12.5 mg/kg) and had a 40%–60% survival rate compared to 5% survival in the untreated group. LPS+ESO-treated mice were rechallenged with LPS of which 80% survived compared to 0% in the control mice. ESO-treated mice had significantly lower serum TNF-α, IL-1ß, and MΦs. The TLR agonism was also not specific for TLR4 as TNF-α and IL-1ß secretion was similarly reduced when stimulated with zymosan, a TLR2 agonist. The impact of ESO on non-infectious pathology was also assessed using sodium thioglycolate-induced peritonitis wherein mice injected with thioglycolates showed reduced infiltrating cells when compared to mice that received only thioglycolate. Thioglycolate+ESO mice also demonstrated reduced levels of macrophage inflammatory protein (MIP)-2 expressing neutrophils and monocyte chemoattractant protein (MCP)-1 expressing MΦs.

Sun et al. (31) treated human PBMCs *in vitro* with the prodrug OME 12.5–50 µg/mL for 4–24 h to assess cytokine production, phagolysosomal acidification, and transcriptomic analysis. OME inhibited TNF-α, MCP-1, IL-12, and IL-23 and the acidification of phagolysosomes. OME changed the transcriptional profile specifically of genes related to TLR signaling and cytokine signaling pathways. OME, LAN, PAN, and rabeprazole (RBP) inhibited the activation of nuclear factor (NF)-κB through suppression of the TLR4 signaling pathway. OME TLR4 inhibition was related to endosomal acidification specifically attenuating TLR signaling endosomes. This inhibition of TLR signaling was conserved across other endosomal signaling TLRs including TLR3 and TLR7/8 while having no effect on pathways activated by extracellular receptors TLR1/2. Taken together, these studies show PPI treatment altering signaling pathways, particularly TLR signaling, and altering p38 phosphorylation patterns.

### PPIs alter neutrophil and macrophage gene expression and cell markers

Schulz-Geske et al. (32) used murine bone marrow-derived MΦs exposed to LAN 5–100 µM for 8 h to assess the expression of heme oxygenase (HO)-1 mRNA, ferritin mRNA, and ROS. LAN increased HO-1 mRNA, protein expression, ferritin, and ROS generation. Though HO-1 is induced by oxidative stress, preincubation with superoxide dismutase to alleviate oxidative stress did not reduce the increase in HO-1 expression. Ubagai et al. (33) isolated PMNs from healthy donors and then co-incubated the cells with LAN 0–10 µg/mL and LPS for 3 h. LAN had a negligible impact on cell viability. Chemokine receptor (CXCR)1/2, CD14, TLR4, TNF-α, and CD11b/CD18 mRNA transcripts were decreased in a dose- and time-dependent manner. Zhou et al. (34) treated wild-type C57/BL6 mice OME or ESO 10 mg/kg daily for 3 weeks prior to ischemic-reperfusion surgery. ESO but not OME caused a marked reduction in IL-1 and CD86 expression, but neither OME nor ESO changed the expression of CD206. Taken together, PPI displayed inhibitory effects on neutrophils and MΦs, especially M1 macrophages.

## Discussion

**Figure 2:**
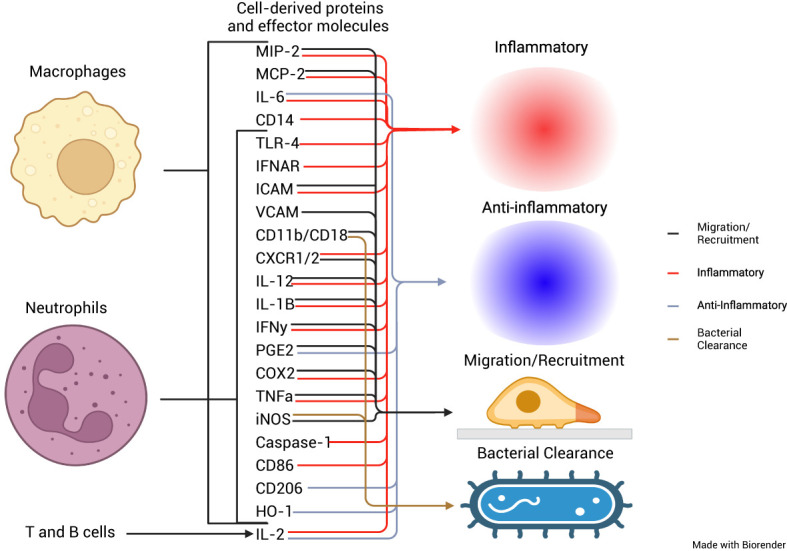
Cellular origins of cytokines and effector proteins and their roles in the immune response. Schematic representation of the cellular origins of multiple different cytokines affected by PPIs, from macrophages, neutrophils, and T and B cells and their roles in either proinflammation, anti-inflammation, migration and recruitment, or bacterial clearance.

**Figure 3:**
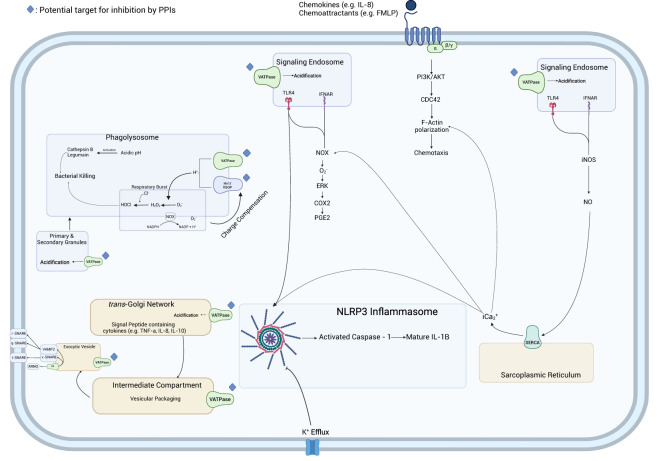
Network of PPI-impacted immunocyte activities. In the phagolysosome, PPIs impact the influx of H^+^ ions driven by the V-ATPase, playing a role in the generation of oxidative radicals by NADPH oxidase and the acidic environment necessary for the activation of cathepsin B and legumain. Furthermore, they inhibit ROS generation in the phagolysosome by interfering with the charge compensatory effect of Hv1/VSOP preventing membrane depolarization which inhibits NADPH oxidase and prevents the generation of ROS. PPIs inhibit the acidification of the endosome necessary for signal transduction from signaling endosomes. The inhibition of calcium release from the sarcoplasmic reticulum inhibits the generation of O_2_^−^ by NOX preventing the stimulation of ERK and COX2 and the generation of PGE2 as well as inhibiting F-actin polymerization. The inhibition of K^+^ efflux by PPIs inhibits the activation of the NLRP3 inflammasome and subsequent activation of caspase-1 which generates mature IL-1ß allowing for its secretion. PPIs inhibit the secretion of the signal peptide-containing cytokines by preventing the acidification of the *trans-*Golgi network and secretory vesicles and inhibiting the proper interaction of V_0_ V-ATPase subunit and SNARE proteins.

In this review, we summarized available literature on the impact of PPIs on MΦs and neutrophils and the immune response to bacterial pathogens. The effects that PPIs exerted on PMNs and MΦs occurred due to the therapeutic mechanism of PPIs, the protonation of sulfhydryl groups, and the subsequent formation of a disulfide bond, while others occurred in the absence of protonation. These findings indicate the propensity of PPIs to act pleiotropically within the cell. The inhibition of cytokine production from neutrophils or macrophages was most studied. Cytokine functions included inflammatory and migration/recruitment along with anti-inflammatory and bacterial clearance (**Figure 2**). A proposed schematic for the PPI pleotropic properties to affect these and other changes is shown in **Figure 3** providing a mechanism between PPI inhibition of V-ATPases and downstream effects in macrophages and neutrophils. A unifying theme is the inhibition of acidification of lysosomal and vacuolar spaces by V-ATPases within the cell. In the phagolysosome, this blocking of acidification is associated with inhibition of NOX activity; reduced bactericidal compounds, H_2_O_2_, HOCl, cathepsin B, and legumain inactivation; and blocked degranulation leading to reduction in bactericidal clearance. This anti-acidifying effect may take place in the Golgi apparatus and provides a potential explanation for the decrease in signal peptide containing cytokines such as IL-2 (35), IL-12 (36), and IFN-γ (37). Packaging of proteins into intermediary vesicles is an ATPase-dependent function (38), and inhibition of granular pH inhibits exocytosis of secretory granules (39). This acidification allows for a dissociation of the V_0_ subunit from the V_1_ of the V-ATPase allowing it to interact with v-soluble *N-*ethylamide-sensitive factor attachment protein (SNAP) receptor (SNARE) and vesicle-associated membrane protein (VAMP) 2 (40), which leads to exocytosis by interacting with t-, q-, and r-SNAREs (41).

This inhibition of acidification in the signaling endosome blocked signal transduction from interferon-alpha/beta receptor (IFNAR) and TLR4, two receptors critical to generating immune responses to bacterial infections. The maturation of these TLRs in the endosome requires acidification to activate proteases which cleave and generate mature signal-transducing TLRs (42). This process is driven by ATP6V0D2, a vesicular H^+^ pump, which plays a role in other important pathways in the macrophage (43) and is inhibited by pantoprazole in other cell types (44–46). Similarly, there was a potential association between the observed inhibition of cytoplasmic Ca^2+^ (18, 19), and these anti-IFNAR/TLR signaling effects may be through the inhibition of iNOS (47, 48), which stimulates the production of NO and the release of Ca^2+^ from the endoplasmic reticulum (ER) by sarcoendoplasmic reticulum calcium ATPase (SERCA). The inhibition of secreted IL-1ß observed in Hinoki et al. (49), Balza et al. (30), and Haas et al. (21) may be associated with decreased efflux of Ca^2+^ which is critical (50, 51) to generate mature IL-1ß (52). This inhibition of Ca^2+^ efflux could be a compounding factor for the inhibition of PGE2 seen in Nakagawa et al. (15) and Haas et al. (21) as Ca^2+^ (53, 54).

Another result worth noting is the conflicting findings regarding the cytotoxicity of PPIs on macrophages and neutrophils. Capodicasa et al. reported that caspase-3-dependent apoptosis of immune cells was increased following a 10-h incubation period, which was capable of being inhibited by a caspase-3 and caspase-8 inhibitor, dependent on the specific PPI used. This study was the only one that reported any significant cytotoxic effects of a PPI. While this may be explained by the longer exposure duration, it is worth noting that in Schulz-Geske et al., there was a reported increase in the expression of HO-1, a protein which can protect monocytes from caspase-3-induced apoptosis (55).

In summary, the lipophilic structure of proton pump inhibitors allows them to easily traverse the cell membrane where, once in the cell, they have the potential to traverse vacuolar membranes and accumulate within acidic intracellular environments. In this review, we have shown evidence that PPIs interfere with macrophage and neutrophil cell function and many of these findings show the canonical action of PPIs (binding and inhibiting H^+^ proton pumps). The inhibition of these vacuolar ATPases by PPIs is likely both dose- and time-dependent as the molecules must first accumulate within these intracellular spaces to a degree sufficient to inhibit these proton pumps. Furthermore, the activity of the prodrug form of PPIs was not assessed in this review. The off-target effects of the prodrug forms of PPIs have become a subject of study as of late particularly in regard to the association of PPIs with Alzheimer’s dementia due to *in-silico* and *in-vitro* findings showing these prodrug PPIs bind and inhibit the synthesis of acetylcholine (56, 57). These findings have been recapitulated in live cell models where it was shown that esomeprazole sufficiently inhibited acetylcholine synthesis in sperm cells (58). Given the role of the cholinergic anti-inflammatory pathway in the regulation of anti-inflammatory cytokines such TNF-α (59), a molecule repeatedly found to be inhibited by PPIs, and the expression of acetylcholine synthesizing ChAT in certain subsets of macrophages (60), it is likely that the prodrug form of PPIs plays a role in the dysregulation of the innate immune response discussed herein. Given the proclivity of healthcare professionals to overprescribe and patients to overuse PPIs, these results highlight the necessity for guidelines on optimizing PPI use and stewardship to curtail unnecessary drug use.

The association between long-term PPI use and infectious diseases and the recent findings that long-term PPI use is associated with increased morbidity and mortality among the critically ill highlight that PPIs may need to be curtailed and alternative treatments for GERD may be needed. While the immunomodulatory effects of PPIs have been shown *in vitro*, the effects of these *in vivo* including human studies are needed. A potential anticholinergic effect of PPIs may provide another immunomodulatory pathway for these drugs to exert change, and investigations into the effects these changes have on the microbiome may provide further insight into their association with increased infection risk and worse patient outcomes.

## Data Availability

The original contributions presented in the study are included in the article/**Supplementary Material**. Further inquiries can be directed to the corresponding author.
