# Supplementary material for: Proton pump inhibitor effect on macrophage and neutrophil function: a systematic review
Source: Front Immunol. 2024 Dec 24;15:1477993. doi: 10.3389/fimmu.2024.1477993 (PMC11703997; doi:10.3389/fimmu.2024.1477993)
Supplement: Supplementary file 1 [file Table1.docx]

Supplemental Table 1. Manuscripts identified for this review

| Reference Number | Authors | Year Published | Journal | Title | PPI |
| --- | --- | --- | --- | --- | --- |
| 11 | J. H. Wandall | 1992 | Gut | Effects of omeprazole on neutrophil chemotaxis, super oxide production, degranulation, and translocation of cytochrome b-245 | Omeprazole |
| 12 | M. Suzuki, M. Mori, S. Miura, M. Suematsu, D. Fukumura, H. Kimura, et al. | 1996 | Free Radical Biology and Medicine | Omeprazole attenuates oxygen-derived free radical production from human neutrophils | Omeprazole |
| 26 | M. Ritter, P. Schratzberger, H. Rossmann, E. Wöll, K. Seiler, U. Seidler, et al. | 1998 | The British Journal of Pharmacology | Effect of inhibitors of Na+/H+-exchange and gastric H+/K+ ATPase on cell volume, intracellular pH and migration of human polymorphonuclear leucocytes | Omeprazole |
| 13 | E. Capodicasa, F. De Bellis, M. A. Pelli | 1999 | Immunopharmacology and Immunotoxicology | Effect of lansoprazole on human leukocyte function | Lansoprazole |
| 27 | T. Ohara, T. Arakawa | 1999 | Digestive Diseases and Sciences | Lansoprazole decreases peripheral blood monocytes and intercellular adhesion molecule-1-positive mononuclear cells | Lansoprazole |
| 20 | G. Agastya, B. C. West, J. M. Callahan | 2000 | Immunopharmacology and Immunotoxicology | Omeprazole inhibits phagocytosis and acidification of phagolysosomes of normal human neutrophils in vitro | Omeprazole |
| 28 | N. Yoshida, T. Yoshikawa, Y. Tanaka, N. Fujita, K. Kassai, Y. Naito, et al. | 2000 | Alimentary Pharmacology & Therapeutics | A new mechanism for anti-inflammatory actions of proton pump inhibitors--inhibitory effects on neutrophil-endothelial cell interactions | Lansoprazole and Omeprazole |
| 14 | K. Zedtwitz-Liebenstein, C. Wenisch, S. Patruta, B. Parschalk, F. Daxböck and W. Graninger | 2002 | Critical Care Medicine | Omeprazole treatment diminishes intra- and extracellular neutrophil reactive oxygen production and bactericidal activity | Omeprazole |
| 18 | O. Handa, N. Yoshida, N. Fujita, Y. Tanaka, M. Ueda, T. Takagi, et al. | 2006 | Inflammation Research | Molecular mechanisms involved in anti-inflammatory effects of proton pump inhibitors | Lansoprazole and Omeprazole |
| 47 | A. Hinoki, K. Yoshimura, K. Fujita, M. Akita, R. Ikeda, M. Nagashima, et al. | 2006 | Pediatric Surgery International | Suppression of proinflammatory cytokine production in macrophages by lansoprazole | Lansoprazole and Omeprazole |
| 19 | R. Martins de Oliveira, E. Antunes, J. Pedrazzoli, Jr. A. Gambero | 2007 | Inflammation Research | The inhibitory effects of H+ K+ ATPase inhibitors on human neutrophils in vitro: restoration by a K+ ionophore | Pantoprazole |
| 9 | E. Capodicasa, P. Cornacchione, B. Natalini, A. Bartoli, S. Coaccioli, P. Marconi, et al. | 2008 | International Journal of Immunopathology and Pharmacology | Omeprazole induces apoptosis in normal human polymorphonuclear leucocytes | Omeprazole |
| 32 | S. Schulz-Geske, K. Erdmann, R. J. Wong, D. K. Stevenson, H. Schröder, N. Grosser | 2009 | World Journal of Gastroenterology | Molecular mechanism and functional consequences of lansoprazole-mediated heme oxygenase-1 induction | Lansoprazole and Omeprazole |
| 33 | T. Ubagai, Y. Koshibu, O. Koshio, T. Nakaki, Y. Ono | 2009 | Journal of Infection and Chemotherapy | Downregulation of immunomodulator gene expression in LPS-stimulated human polymorphonuclear leukocytes by the proton pump inhibitor lansoprazole | Lansoprazole |
| 15 | S. Nakagawa, Y. Arai, T. Kishida, N. Hiraoka, S. Tsuchida, H. Inoue, et al. | 2012 | Inflammation | Lansoprazole inhibits nitric oxide and prostaglandin E(2) production in murine macrophage RAW 264.7 cells | Lansoprazole |
| 33 | E. Balza, P. Piccioli, S. Carta, R. Lavieri, M. Gattorno, C. Semino, et al. | 2016 | Cell Death and Disease | Proton pump inhibitors protect mice from acute systemic inflammation and induce long-term cross-tolerance | Omeprazole and Esomeprazole |
| 21 | C. M. Haas, M. Maywald, A. Goetzenich, C. Stoppe and L. Rink | 2018 | Journal of Leukocyte Biology | Proton-pump inhibitors elevate infection rate in cardiothoracic surgery patients by influencing PMN function in vitro and in vivo | Omeprazole and Pantoprazole |
| 22 | T. Bosnjak, R. Solberg, P. D. Hemati, A. Jafari, M. Kassem, H. T. Johansen | 2019 | Basic and Clinical PHarmacology and Toxicology | Lansoprazole inhibits the cysteine protease legumain by binding to the active site | Lansoprazole |
| 31 | L. Sun, Y. Liu, X. Liu, R. Wang, J. Gong, A. Saferali, et al. | 2022 | Advanced Science | Nano-Enabled Reposition of Proton Pump Inhibitors for TLR Inhibition: Toward A New Targeted Nanotherapy for Acute Lung Injury | Omeprazole |
| 34 | G. Zhou, Y. Peng, M. Guo, C. Qu, S. Luo, Y. Jiang, et al. | 2022 | Biochemical and Biophysical Research | Esomeprazole inhibits endoplasmic reticulum stress and ameliorates myocardial ischemia-reperfusion injury | Esomeprazole |
